# The characterization and manipulation of the bacterial microbiome of the Rocky Mountain wood tick, *Dermacentor andersoni*

**DOI:** 10.1186/s13071-015-1245-z

**Published:** 2015-12-10

**Authors:** Katie A. Clayton, Cory A. Gall, Katheen L. Mason, Glen A. Scoles, Kelly A. Brayton

**Affiliations:** School of Molecular Biosciences, Washington State University, Pullman, WA 99164-7010 USA; Department of Veterinary Microbiology and Pathology, Paul G. Allen School for Global Animal Health, Washington State University, Pullman, WA 99164-7040 USA; Animal Disease Research Unit, U.S. Department of Agriculture, Agricultural Research Service, Washington State University, Pullman, WA 99164-6630 USA

**Keywords:** Ticks, Microbiome, Endosymbiont

## Abstract

**Background:**

In North America, ticks are the most economically impactful vectors of human and animal pathogens. The Rocky Mountain wood tick, *Dermacentor andersoni* (Acari: Ixodidae), transmits *Rickettsia rickettsii* and *Anaplasma marginale* to humans and cattle, respectively. In recent years, studies have shown that symbiotic organisms are involved in a number of biochemical and physiological functions. Characterizing the bacterial microbiome of *D. andersoni* is a pivotal step towards understanding symbiont-host interactions.

**Findings:**

In this study, we have shown by high-throughput sequence analysis that the composition of endosymbionts in the midgut and salivary glands in adult ticks is dynamic over three generations. Four *Proteobacteria* genera, *Rickettsia, Francisella, Arsenophonus,* and *Acinetobacter*, were identified as predominant symbionts in these two tissues. Exposure to therapeutic doses of the broad-spectrum antibiotic, oxytetracycline, affected both proportions of predominant genera and significantly reduced reproductive fitness. Additionally, *Acinetobacter*, a free-living ubiquitous microbe, invaded the bacterial microbiome at different proportions based on antibiotic treatment status suggesting that microbiome composition may have a role in susceptibility to environmental contaminants.

**Conclusions:**

This study characterized the bacterial microbiome in *D. andersoni* and determined the generational variability within this tick. Furthermore, this study confirmed that microbiome manipulation is associated with tick fitness and may be a potential method for biocontrol.

## Findings

### Background

The presence of diverse microbial communities within multicellular organisms has been well documented, consisting of bacteria, protozoans, filarial nematodes, fungi, and viruses [1, 2]. These microbes have formed symbiotic relationships with their hosts, which can be commensal, mutualistic, or parasitic [3]. Symbiont literature defines primary endosymbionts as having an obligate relationship essential for the fitness of the host, whereas secondary symbionts are microbes that have likely become associated via horizontal transmission through environmental exposure [4–6]. Although secondary symbionts are less likely to provide an essential benefit to the host, their function within the microbiome and benefit to the host is not fully understood [7, 8]. On an ecological level, the microbiome is an active community that involves symbiont-symbiont interactions as well as symbiont-host communication. Primary and secondary endosymbiont theory is well documented for insect hosts, however it is unknown if these relationships hold for ticks.

Hard ticks (*Acari*: *Ixodidae*) are among the world’s leading vectors of diseases in humans and livestock and are second only to mosquitos in overall impact on global health [9, 10]. The Rocky Mountain wood tick, *Dermacentor andersoni,* transmits a number of pathogens including the etiological agents of Rocky Mountain spotted fever and bovine anaplasmosis. The three genera of bacterial endosymbionts that have been associated with *D. andersoni* ticks include: *Arsenophonus*, *Francisella*, and *Rickettsia* [7, 11–14]. While forays into high throughput characterization of the bacterial microbiome in ticks have been undertaken in recent years, the composition of the microbiome in *D. andersoni*, including the microbial associations within the midgut and salivary glands (the relevant organs for acquisition and transmission of pathogens) remains unknown [1, 15].

Treatment with antibiotics has previously been shown to be a useful approach to study the roles of endosymbionts within their hosts [4, 16]. A recent study showed that direct injection of tetracycline or rifampicin into *Amblyomma americanum*, reduced the bacterial load of a *Coxiella* species, an endosymbiont known to be trans-stadially transmitted, and led to reduced reproductive fitness [4]. While this study did not involve a generational analysis, a reduction in the number of larvae that survived in the treated groups resulted in fewer ticks that survived to reproductive maturity.

The stability in the composition of both core and minor endosymbionts varies between systems; though there have been a growing number of tick microbiome studies [17, 18], little is known about the microbiome of *D. andersoni*, and specifically if it is possible to manipulate the microbiome of this species. Is the composition of the endosymbiont population of *D. andersoni* stable over time? Is it possible to manipulate the bacterial microbiome of *D. andersoni* through antibiotic treatment and will this affect the fitness of the tick? To address these questions, we employed a high-throughput sequencing approach to characterize the bacterial microbiome of two *D. andersoni* tissues, the midgut (MG) and salivary glands (SG) over three generations (T1, T2, and T3) as well as studying the effects of antibiotic treatment on the microbiome.

## Methods

### Ethical approval

Tick rearing and animal experiments were carried out under guidelines approved by the Institutional Animal Care and Use Committee (Washington State University IACUC #04440-004 and University of Idaho IACUC #2013-66).

### Experimental design

**Fig. 1:**
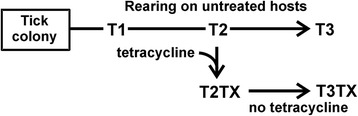
Experimental design. A cohort of ticks was sampled from the laboratory colony (designated T1). The colony was reared for 1 generation and then split into 2 cohorts; one cohort was exposed to oxytetracycline during feeding (designated T2TX) and the other was fed on an untreated calf (designated T2). Following exposure, each group of ticks were reared one generation and fed as adults on untreated calves prior to dissection (designated T3 and T3TX)

The USDA, ARS Animal Disease Research Unit located at the Holm Research Center at the University of Idaho in Moscow, Idaho maintains a colony of *D. andersoni* ticks that originated from Reynolds Creek, Idaho (RC). Larvae were fed using Sprague–Dawley rats and nymphs and adults were fed on Holstein cattle. The bacterial microbiome of adult male ticks, which are the principle vector of *Anaplasma marginale*, was characterized over three generations (T1-T3 cohorts, Fig. 1). One male cohort from the T2 generation was fed on an untreated (normal) calf, while the second cohort was exposed to oxytetracycline by allowing them to feed on an antibiotic-treated calf. The antibiotic-treated calf was subcutaneously dosed with 11 mg/ kg body weight of Liquamycin (LA-200, Zoetis, Florham Park, NJ) on days −4, −1, 3, and 7-days post-infestation, which was a therapeutic dosing regime that might be used to treat an infection. Male ticks from this group were fed for 7 days and then MG and SG were dissected. A colony of ticks was established from the treated cohort to follow the microbiome for two generations (T2TX and T3TX).

For characterization of the microbiome, 90 fed male ticks from each treatment group were surface sterilized by rinsing in 5 % EtOH and 5 % bleach water solution, followed by a double rinse in H_2_0. The MG and SG were then dissected and pooled in groups of 30 ticks with three biological replicates, with each biological replicate analyzed in triplicate. Genomic DNA was isolated using the PureGene Extraction Kit (Qiagen, Germantown, MD). PCR amplification was performed using barcoded sample-specific primers [19] targeting variable region 4 of the 16S ribosomal DNA gene (primers E517F and E806Rm) and the PCR products submitted for Roche 454 GS FLX Titanium pyrosequencing [1]. The resulting reads were processed in CLC Genomics Workbench, Black Box Chimera Check (B2C2), and RDP Classifier [20, 21]. Sequence reads below a Phred score threshold of 20 and shorter than 100 bases were discarded. Sequence data was deposited in the sequence read archive at NCBI (SRP063991). PCR assays were employed for the major endosymbionts to discriminate at the species level: *rOmpA* for *Rickettsia* (primers Rr190.70p and Rr190.602n) [22] and 16S rDNAfor *Francisella* (primers 61 F and 1227 F) [23].

In order to determine if microbiome manipulation through antibiotic exposure influenced the fitness of ticks, we measured several fitness parameters of the T3 and T3TX colonies (Table 1). The colonies were subsampled and the parameters that were analyzed included: larval survival, fed larval weight, larva-nymph molt, nymphal feeding success, fed nymphal weight, and nymph-adult molt. Pair-wise comparisons were used to determine fitness differences between groups using SPSS (IBM V. 20).

**Table 1:** Reproductive fitness of offspring of *D. andersoni* ticks from T3 and T3TX colonies

	Untreated Ticks (T3)	Treated Ticks (T3TX)
Total larvae at start	3000	3000
Larval survival	40 %	15 %**
Mean fed larval weight	0.521 mg	0.487 mg*
Larva-nymph molt	83.7 %	77.8 %*
Total nymphs	908	271
Nymphal feeding success	50.7 % (460/908)	42.4 % (116/271)
Mean fed nymphal weight	11.7 mg	11.4 mg
Nymph-adult molt	97.4 %	98.3 %
Total adults	448	114

*p <0.05;**p < 0.001

## Results and discussion

**Fig. 2:**
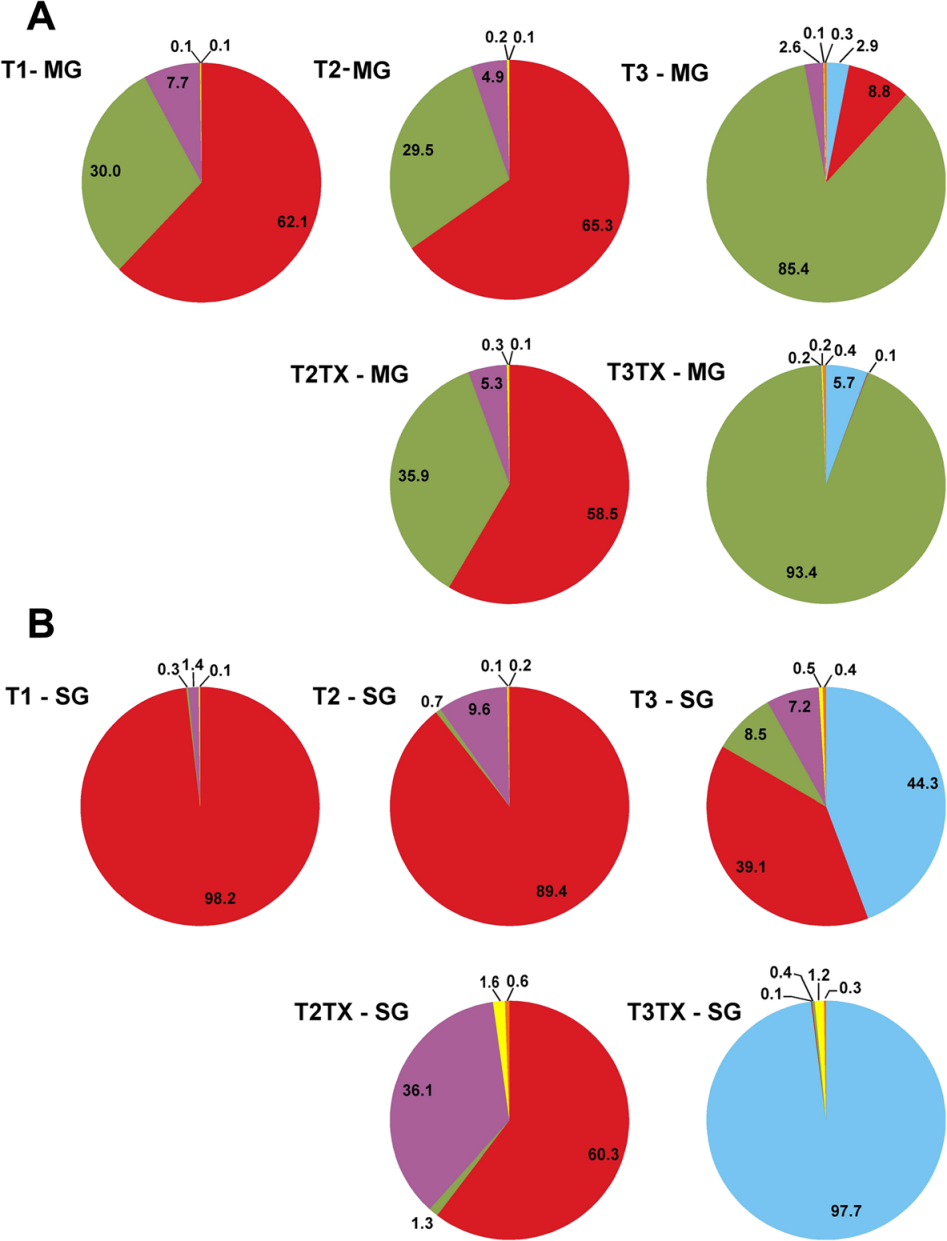
Microbiome composition of the midgut and salivary glands of adult male ticks over time and in response to antibiotics. **a** The microbiome of the midgut (MG) and **b** the microbiome of the salivary glands (SG). Predominant genera include *Acinetobacter* (*light blue*), *Arsenophonus* (*red*), *Francisella* (*green*), and *Rickettsia* (purple)*.* Minor genera are represented by Other (*yellow*) and Unclassified reads are represented in orange. T1, T2, and T3 refer to the generation of the ticks after sampling the colony. T2TX refers to the cohort of T2 ticks that were exposed to oxytetracycline while T3TX are the progeny of these ticks

This study presents an in-depth characterization of the bacterial microbiome of *D. andersoni.* Sequencing of 16S rDNA allowed us to elucidate the composition of the bacterial microbiome of MG and SG in male ticks over three generations, as well as how antibiotic exposure alters the microflora (Fig. 1). Using high-throughput pyrosequencing, we obtained a mean of 47,554 reads/sample with an average length of 288 bp/read. Our results showed that the predominant taxa of bacteria in MG were *Arsenophonus*, *Francisella*, and *Rickettsia* in T1 and T2 populations, with *Acinetobacter*, an environmental contaminant, becoming a predominant taxon in T3 (Fig. 2). In SG, the predominant taxa included *Arsenophonus* and *Rickettsia* in T1 and T2 cohorts with *Acinetobacter* and *Francisella* becoming established in T3 (Fig. 2). Proportions of *Arsenophonus* and *Francisella* were relatively stable from T1 to T2 in both MG and SGs, whereas the *Rickettsia* proportion varied between T1 and T2 in both MGs and SGs.

We exposed a cohort of T2 ticks to antibiotics and evaluated the response of the bacterial microbiome as well as tick fitness. Our results showed that the proportions and diversity of bacteria within the microbiome changed when exposed to Oxytetracycline (Fig. 2). Antibiotic exposure caused a 30 % decrease in the relative proportion of *Arsenophonus* in the SGs and resulted in a nearly fourfold increase of *Rickettsia* when compared to the untreated microbiome. In the MGs of treated ticks (T2TX), the proportions of the predominant genera, *Arsenophonus*, *Francisella*, and *Rickettsia*, appear to have only been moderately affected (Fig. 2). Furthermore, *Acinetobacter* proportion expanded in both the MG and SG of both T3 and T3TX. *Acinetobacter* accounted for less than 0.01 % of the microbiome of T2TX, but in the T3 generation, *Acinetobacter* accounted for more than one third of the sequences in T3 SGs and nearly one hundred percent of reads in treated (T3TX) SGs. *Acinetobacter* was also identified in T3 and T3TX MGs but was much less predominant (<6 % of total reads). *Acinetobacter* is a known environmental contaminate and commonly found in soil; thus, a possible source of exposure was the time on the cattle. Although it has been demonstrated that the host does not influence the bacterial microbiome of ticks [17, 24], there has yet to be a study analyzing the impact of the host’s microbial community at the feeding site.

Interestingly, the response to *Acinetobacter* invasion was different in treated ticks when compared to untreated ticks, with T3TX ticks having about twice as much *Acinetobacter* as T3 ticks, regardless of tissue. This suggests that vulnerability to invading endosymbionts varies, perhaps by limiting factors within the microbiome that affect bacterial interaction. These limiting factors may be physical space, nutrient availability, or a more direct cause, such as a bacterium producing a deterrent. Disrupting the microbiome appears to have resulted in an increased susceptibility to *Acinetobacter*.

Because species level discrimination was not possible with 454 pyrosequencing we employed PCR assays for *Rickettsia* and *Francisella* classification. Using *rOmpA* primers identified *R. peacockii* and *R. rhipicephali* in all MG samples, with the exception of T3TX. *R. rhipicephali* was found in all SG samples except T3TX. Furthermore, *R. rhipicephali, R. philipii, R. peacockii,* and *R. marmionii* were found in both treated and untreated MG and SG samples, with *R. bellii* identified only in T2TX-MG. In both MG and SG samples, the *Francisella*-like endosymbiont (FLE) previously detected in both *D. andersoni* and *D. occidentalis* (GenBank accession no. AY375397.1 and AY375402.1, respectively) was identified. These findings are consistent with previous studies that have shown that *D. andersoni* ticks are colonized by rickettsial endosymbionts [13, 25] in addition to FLE [7, 12] and *Arsenophonus* [11]. These genera of bacteria are known to be transmitted transovarially and thus fit the definition of primary endosymbionts.

The second aspect of the antibiotic exposure study was to examine reproductive fitness of *D. andersoni* to see if there was an association between the changes in the microbiome and fitness. We saw significant reductions of fitness in the treated tick colony (T3TX) at several life stages (Table 1). There was a 25 % lower larval survival after feeding in T3TX ticks as compared to the untreated (T3) group (*p < 0.01*). Mean larval weight after feeding was significantly reduced in T3TX ticks (*p < 0.05*) as was the survival after the larva-nymphal molt (*p < 0.05*). While there was no significant decrease in mean nymphal weight or nymph to adult molt, the total number of treated ticks surviving to adulthood was 25 % that of untreated ticks (Table 1). Antibiotic-exposed ticks were less competent at survival, feeding, and molting. Our results support findings in *A. americanum* ticks where there was significant reduction in fitness post-antibiotic exposure [4]. Although it is unclear how the observed changes in the microbiome could impact fitness, the sequence data indicates that antibiotic exposure had a measurable impact on the microbiome.

## Conclusions

This study has identified the major endosymbionts found in *D. andersoni* ticks and demonstrates microbiome volatility over time. Additionally, we have shown that the microbiome can be influenced by antibiotic exposure, and that these changes vary by tissue. Our findings suggest that disturbance to the microbiome may result in an increased vulnerability to microbial invasion by environmental microbes. These conclusions are the first steps in understanding the relationships between endosymbionts and *D. andersoni* and these data suggest that further investigation into the function of the microbiome is warranted [4–6].

## Abbreviations

T1-T3: generation 1, 2, and 3
T2TX: generation 2 and exposed to antibiotics
T3TX: generation 3, progeny of antibiotic-exposed ticks
MG: midgut
SG: salivary glands
